# Supplementary material for: Scalable, High‐Throughput Isolation of Extracellular Vesicles Using Electrokinetic‐Assisted Mesh Filtration: ExoFilter
Source: J Extracell Biol. 2025 May 28;4(6):e70054. doi: 10.1002/jex2.70054 (PMC12119903; doi:10.1002/jex2.70054)
Supplement: Supplementary file 1 — Figure S1. Full western blot image corresponding to Figures 2g and 5e. Figure S2. Impact of 1 M NaCl and PBS on EV size and concentration. Figure S3. Cytotoxicity test and wound healing assay. Figure S4. Accelerated ageing test results at different temperature demonstrating long‐term mesh stability. Figure S5. BCA standard curve. [file JEX2-4-e70054-s001.docx]

**Scalable, high-throughput isolation of extracellular vesicles using electrokinetic-assisted mesh filtration: ExoFilter**

KangMin Lee^1^, Minju Bae^1^, YongWoo Kim^1^, SoYoung Jeon^2^, Sujin Kang^4^, Wonjong Rhee^4*^, Sehyun Shin^1,2,3*^

^1^ School of Mechanical Engineering, Korea University, Seoul, Republic of Korea

^2^ Department of Micro-Nanosystem Technology, Korea University, Seoul, Korea

^3^ Engineering Research Center for Biofluid Biopsy, Seoul, Korea

^4^ Department of Bioengineering and Nano-Bioengineering, Incheon National University, Incheon, Republic of Korea

* To whom correspondence should be addressed:

Sehyun Shin, Ph.D.

Professor,

School of Mechanical Engineering, Korea University, Seoul 02841, Republic of Korea; Tel.: +82 2 3290 3377; Fax: +82 2 928 5825; E-mail: lexerdshin@korea.ac.kr

ORCID ID: 0000-0002-2611-5610


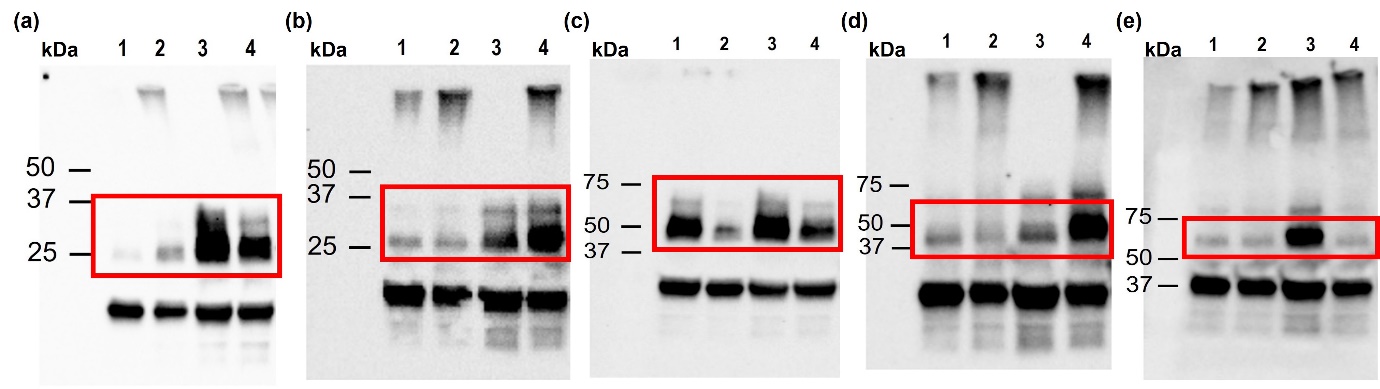


**Figure S1.** Full western blot image corresponding to Figure 2g and 5e. The blot includes four lanes, representing UC, TFF, ExoPAS, and ExoFilter from lane 1 to lane 4, respectively. EVs were isolated from plasma using different extraction methods. Red boxes highlight the positions of the corresponding proteins: (a) CD9, (b) CD81, (c) TSG101, (d) Alix, and (e) Albumin.


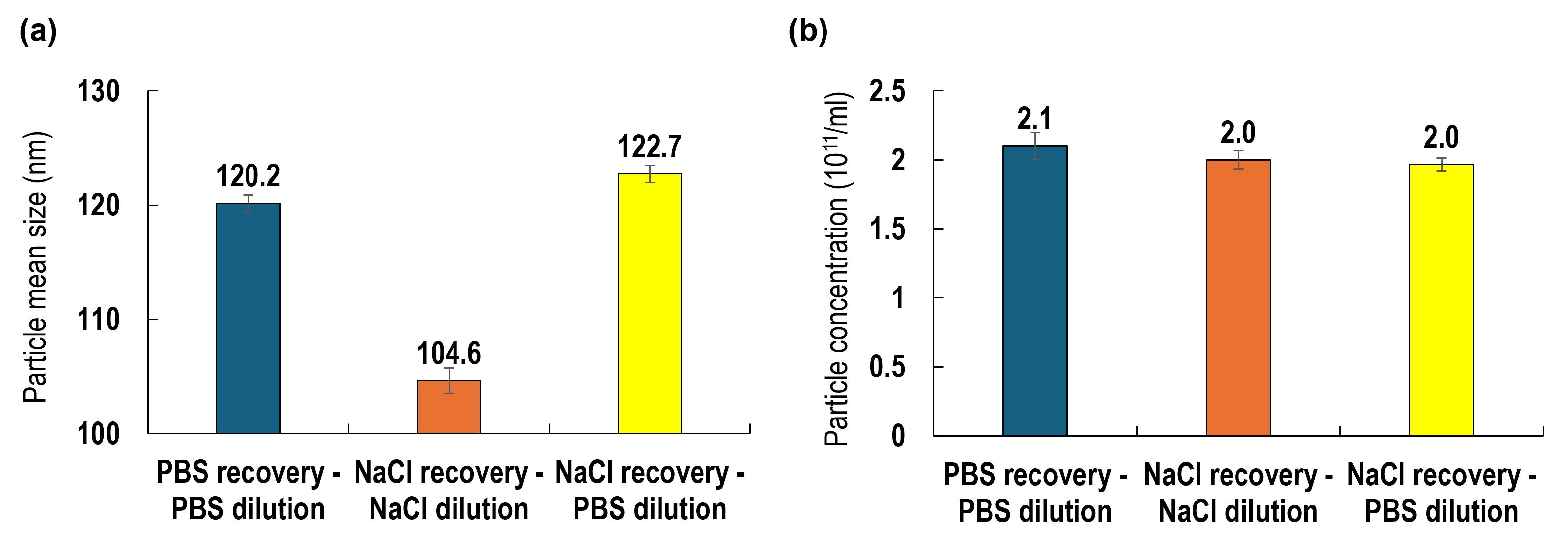


**Figure S2. Impact of 1 M NaCl and PBS on EV size and concentration.** (a) Particle mean size, (b) Particle concentration.

This figure illustrates the impact of 1 M NaCl and PBS as recovery buffers on the size and concentration of extracellular vesicles (EVs). EVs were recovered using two different buffers (1 M NaCl and PBS) to evaluate the effects of hyperosmotic conditions on EV characteristics. The results show that EVs recovered with 1 M NaCl exhibited a reduction in particle mean size to 104.6 nm, compared to 120.2 nm when PBS was used. However, the particle concentration, measured via NTA, remained statistically unchanged at 2.0 × 10¹¹ particles/mL for NaCl and 2.1 × 10¹¹ particles/mL for PBS. To determine whether the size reduction caused by NaCl could be reversed, the EVs were diluted with PBS prior to measurement. Interestingly, the particle mean size recovered to 122.7 nm, while the particle concentration remained stable at 2.0 × 10¹¹ particles/mL. These results confirm that the size reduction induced by hyperosmotic conditions is reversible upon dilution with PBS, and the overall EV concentration remains unaffected. This finding highlights the importance of buffer selection in EV recovery and demonstrates that while 1 M NaCl temporarily reduces EV size, this effect does not compromise EV integrity or concentration.


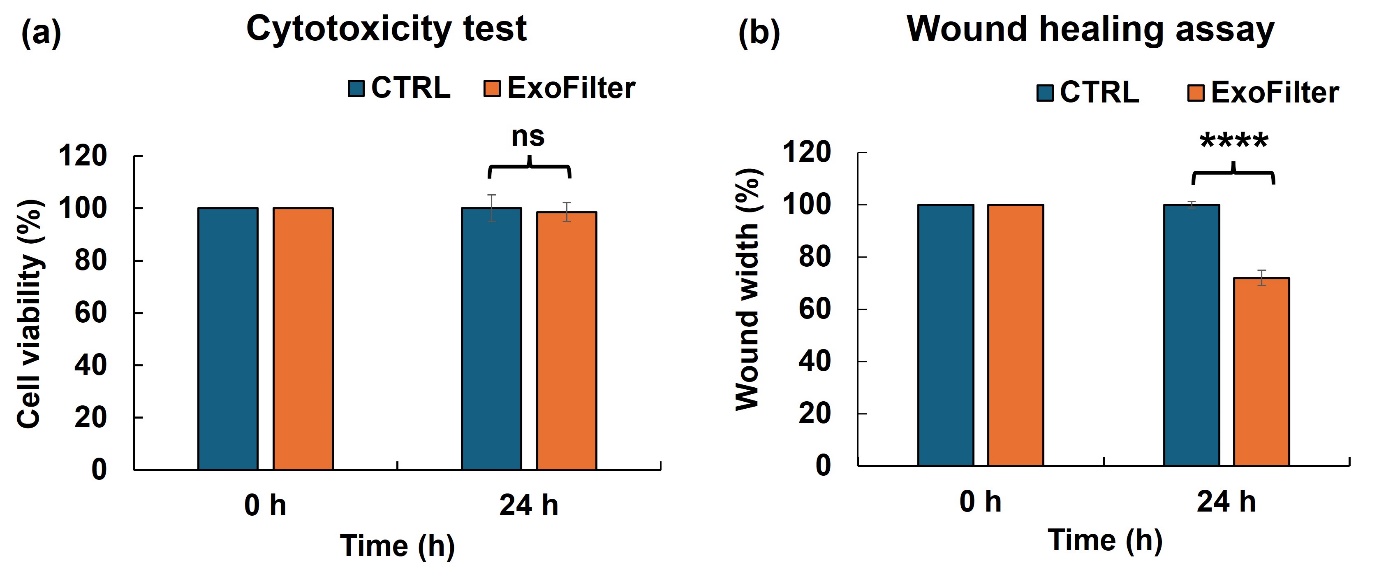


**Figure S3.** Cytotoxicity test and wound healing assay. (^ns^p value is > 0.05, ^****^p value is < 0.0001) We performed cytotoxicity and wound healing assays using EVs isolated from 500 mL of *Centella asiatica* extract (Amicos, Korea) via the ExoFilter bottle-top method under negative pressure (-68 kPa), yielding 100 mL of eluted EVs.

For the cytotoxicity test, HDF cells were cultured, followed by seeding an appropriate number of cells. After 24 hours, the cells underwent 1-hour starvation. The control group was treated with PBS, while the experimental group was treated with EVs isolated from *Centella asiatica* extract (particle concentration = 7.7 × 10^8^ particles/mL). Both groups were incubated for 24 hours, after which cell viability was measured using the CCK-8 assay. The results indicated no cytotoxicity after 24 hours, demonstrating that rapid large-scale EV isolation using negative pressure does not affect the viability of the EVs.

For the wound healing assay, human keratinocytes were cultured and seeded to confluence in petri dishes. After 24 hours, the cells were starved for 1 hour. A scratch was made in the center of each well using a pipette tip, followed by the addition of PBS. Images of the wound areas were captured prior to sample treatment. After that, the control group was treated with PBS, while the experimental group received EVs isolated from *Centella asiatica* extract (particle concentration = 1.5 × 10^8^ particles/mL). Both groups were incubated for 24 hours. After removing the treated EVs and replacing them with PBS, the same wound areas were imaged. Image J software was used to quantify the wound area before (0 h) and after treatment (24 h). The results demonstrated that the EVs had a significant effect on wound healing after 24 hours. This indicates that even EVs isolated rapidly from *Centella asiatica* extract using negative pressure retain their wound healing efficacy.

These two experiments collectively suggest that intact and viable EVs can be obtained efficiently using the ExoFilter bottle-top method under negative pressure, without compromising functionality.


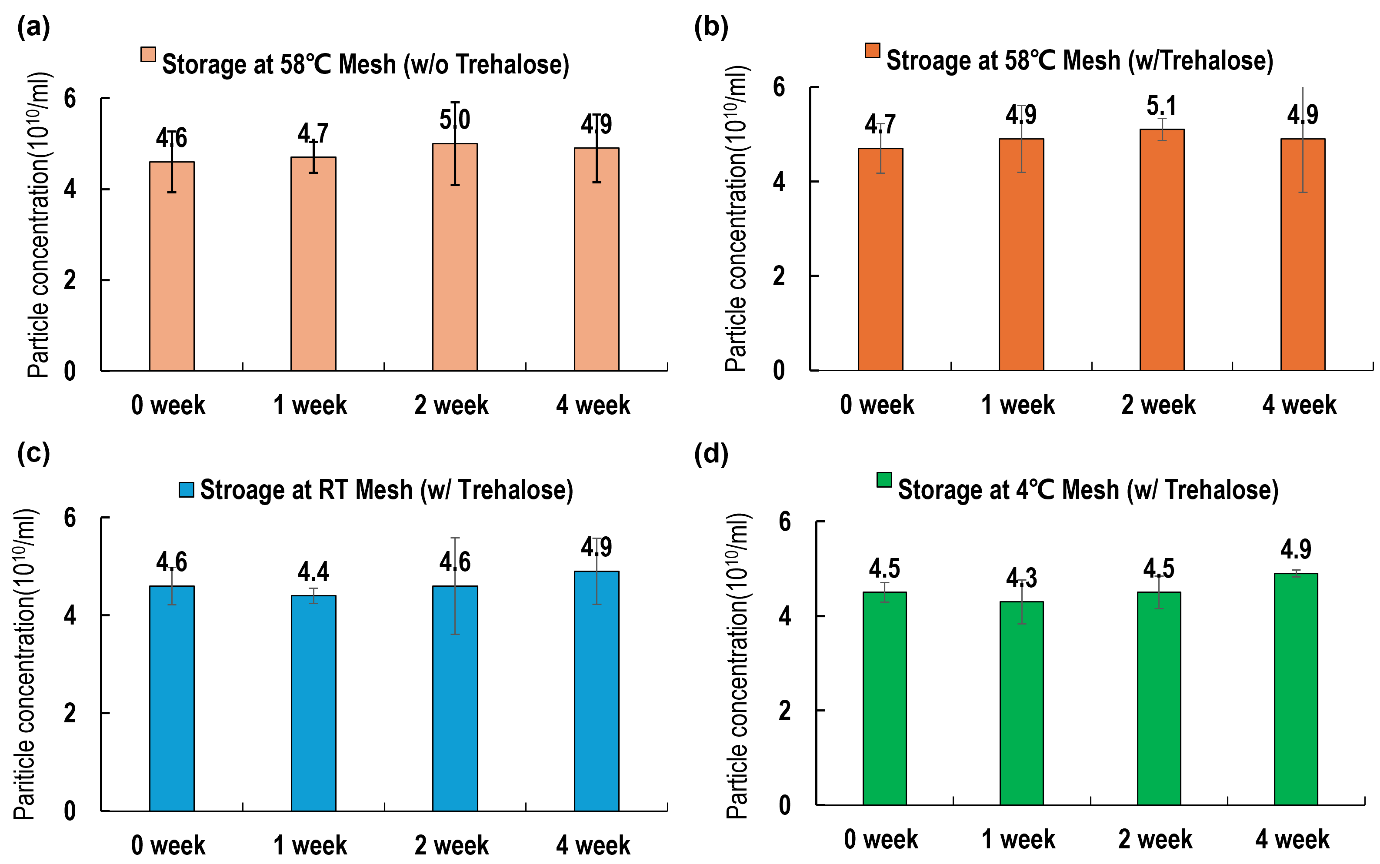


**Figure S4. Accelerated aging test results at different temperature demonstrating long-term mesh stability.** (a) Storage at 58°C Mesh (w/o trehalose). (b) Storage at 58°C Mesh (w/ trehalose). (c) Storage at RT Mesh (w/ trehalose). (d) Storage at 4°C Mesh (w/ trehalose).

This figure shows the results of the accelerated aging test conducted at 58°C to evaluate the long-term stability of the mesh. The test was performed over a 4-week period, and the accelerated aging factor (AAF) was calculated using the following formula: AAF=2^(58−22)/10^=13.9. Based on this factor, the 4 weeks of testing at 58°C correspond to approximately 300 days of real-time aging (28×13.9 ≈ 390 days). The results indicate that the mesh maintained its stability throughout the accelerated aging period, with no significant changes observed in the tested parameters. This confirms the robustness and reliability of the mesh for long-term use under storage conditions.

**Figure S5.** BCA standard curve. The standard curve was used to calculate the protein concentration of the isolated EV samples, providing an estimate of potential contamination levels.
